# Biofunctionalization of HMX with Peptides via Polydopamine Crosslinking for Assembling an HMX@Al@CuO Nanoenergetic Composite

**DOI:** 10.3390/nano13121837

**Published:** 2023-06-10

**Authors:** Miaomiao Jin, Zhanxin Song, Wei Liu, Guozhen Wang, Mo Xian

**Affiliations:** 1 CAS Key Laboratory of Biobased Materials, Qingdao Institute of Bioenergy and Bioprocess Technology, Chinese Academy of Sciences, Qingdao 266101, China; songzx@qibebt.ac.cn (Z.S.); wanggz@qibebt.ac.cn (G.W.); 2 Shandong Energy Institute, Qingdao 266101, China; 3 Qingdao New Energy Shandong Laboratory, Qingdao 266001, China; 4 Institute of Corrosion Science and Technology, Guangzhou 510530, China; liuwei@qibebt.ac.cn

**Keywords:** biological self-assembly, polydopamine, peptide, green preparation, activation energy

## Abstract

Biological approaches for the synthesis of a hybrid explosive–nanothermite energetic composite have attracted greater scientific attention because of their advantages, including their moderate reactions and the absence of secondary pollution. In this study, a simple technique was developed to fabricate a hybrid explosive–nanothermite energetic composite based on a peptide and a mussel-inspired surface modification. Polydopamine (PDA) was easily imprinted onto the HMX, where it maintained its reactivity and was capable of reacting with a specific peptide used to introduce Al and CuO NPs to the surface of the HMX via specific recognition. The hybrid explosive–nanothermite energetic composites were characterized using differential scanning calorimetry (TG-DSC), transmission electron microscopy (TEM), X-ray photoelectron spectroscopy(XPS), and a fluorescence microscope. A thermal analysis was utilized to investigate the energy-release properties of the materials. The HMX@Al@CuO, which benefitted from an enhanced interfacial contact in comparison with the physically mixed sample (HMX-Al-CuO), demonstrated a 41% lower HMX activation energy.

## 1. Introduction

Nanothermites, types of mixtures or compounds that may immediately release enormous amounts of chemical energy via very rapid redox reactions, are widely utilized in both military and civilian applications [1,2,3]. Nanothermites can have high energy densities and low levels of vulnerability, but due to the inherent features of their composition, they have low reaction rates and limited gas yields [4,5]. In contrast, organic, small molecular crystal explosives with high levels of chemical energy typically demonstrate rapid energy releases accompanied by massive gaseous products [6]. Therefore, the integration of nanothermites with explosives will be a complementary technique for enhancing the overall performance of the energetic system. The study by Kaili Zhang demonstrated that the integration of 2,4,6,8,10,12-hexanitro-2,4,6,8,10,12-hexaazaisowurtzitane (CL20) with CuO/Al not only enhanced the overall heat of the reaction and decreased the activation energy by 18.2% [5] but also exhibited a desirable burning behavior with a steady and violent combustion flame. Thermite has a catalytic influence on explosive decomposition. Consequently, the energy release rate and the rate of heat release are mostly governed by the size, the particles’ spatial distribution, and the interface contact between components.

A homogeneous distribution is required in a composite to enhance the combustion performances of nanothermites and explosives. Many efforts [7,8,9,10,11,12,13], including those applying the sol–gel technique, electrostatic assembly, electrophoretic deposition, and self-assembly technique, have been directed toward improving the degree of uniformity of nanothermites, but few studies have attempted to improve the uniformity between thermites and explosives [5,14]. A new method was needed for the preparation of nanothermites in combination with explosives. Dopamine (DA), a molecule inspired by mussels [15], may self-polymerize and attach to nearly all material surfaces under mild conditions without surface pretreatment [16,17,18,19,20]. This property makes DA an attractive choice for the production of energetic materials [17,20,21,22,23]. However, a three-component assembly aided by polydopamine (PDA) has rarely been described. PDA layers containing several catechols and quinones can serve as anchors for grafting diverse biomolecules via Schiff’s base or Michael addition reactions [24,25,26,27]. Previous data from our group demonstrated that the peptide SH-25 (in which the sequences of STEARATTLTACDAY and HHHHHH served as linkers to anchor Al and CuO, respectively) was able to bind Al and CuO NPs and improve the mixing uniformity [28]. Therefore, a strategy to combine PDA coatings with peptides to improve hybrid explosive–nanothermite energetic composites has been proposed.

**Scheme 1:**
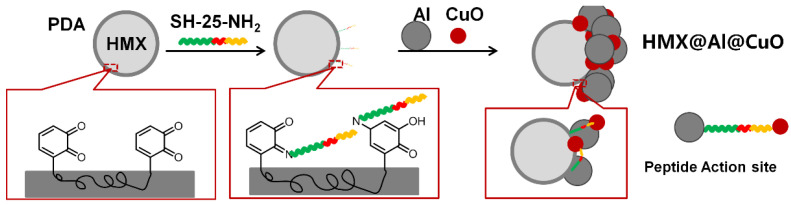
Schematic illustration of the HMX, Al, and CuO assembly process.

HMX (C_4_H_8_N_8_O_8_, 1,3,5,7-tetranittro-1,3,5,7-tetrazocane) is one of the most important nitramine explosives and has been widely used in solid rocket propellants, plastic-bonded explosives (PBXs), and civil explosive materials [22,29]. In the current investigation, an HMX@Al@CuO energetic composite was synthesized. Scheme 1 depicts the PDA–peptide-driven assembly of the HMX, Al, and CuO nanocomposite. First, PDA, which was strongly adhered to the HMX, was produced when DA underwent self-polymerization under moderately alkaline conditions. Two functional groups, quinone and catechol, were presented on the PDA coating, and latent reactivity was transferred toward the quinone groups under alkaline conditions [30]. Then, PDA could be grafting onto the SH-25-NH_2_ peptide via Schiff’s base or Michael addition reactions between the amine and quinone groups. Finally, the recognition sequence of the peptide SH-25-NH_2_ was utilized to introduce Al and CuO NPs onto the surface of the HMX via specific recognition [28]. The Al and CuO nanocomposites were highly dispersed on the HMX crystal surface due to the close interactions between the NPs. Fluorescent studies and X-ray photoelectron spectroscopy (XPS) demonstrated that the peptide was effectively grafted onto the HMX surface. In addition, the thermal characteristics of the energetic composites comprised of HMX@Al@CuO were examined. This approach had the advantages of improved and good adhesion, mixing uniformity, a mild reaction, and environmental friendliness. In addition, this strategy may offer new insights that can be applied to the design of other explosives and nanostructured energetic materials.

## 2. Materials and Methods

### 2.1. Materials

The Nano Material Engineering Company (Jiaozuo, China) provided aluminum NPs with an average diameter of 100 nm and a surface alumina layer thickness of 4.3 ± 0.3 nm. From Shanghai yunfu Nanotechnology Co., Ltd. (Shanghai, China), copper oxide NPs with an average diameter of 20 nm were acquired. 3-Hydroxytyramine hydrochloride (DA-HCl) was acquired from Aladdin Reagent Co. HMX was synthesized in our institute. The phosphate-buffered saline (PBS, 0.1 M, pH 7.0) and tris-HCl (0.1 M, pH 8.5) were acquired from Beijing Solarbio Science & Technology Co., Ltd. (Beijing, China). The Peptides SH-25-NH_2_ (STEARATTLTACDAYGGGGHHHHHH-NH_2_) (purity > 95%) and SK-26 (STEARATTLTACDAYGGGGHHHHHH-K-FITC) (purity > 90%) were acquired from GL Biochem (Shanghai) Ltd. (Shanghai, China).

### 2.2. Methods

The synthesis of peptide-functionalized HMX was as follows. PDA-coated HMX (HMX-PDA) was prepared using the following procedure. First, 30 mg of HMX was dissolved in 10 mL of Tris-HCl solution (10 mM, pH 8.5) and sonicated for 5 min at 100 W using a SCIENTZ-IID ultrasonic probe system, with 2 s pulses separated by 1 s. After that, 30 mg of DA-HCl was added to the HMX solution. Following 30 min of stirring at ambient temperature, the reaction mixture was centrifuged once at 5000 rpm to terminate the reaction, and it was then ultrasonically dispersed in 10 mL of 10 mM Tris-HCl solution (pH 8.5) for future use.

The peptide-functionalized HMX-PDA (HMX-PDA-SH-25-NH_2_) was synthesized in the following manner. A 10 mL aqueous solution of the SH-25-NH_2_ peptide (150 μM in 10 mM Tris–HCl, pH 8.5) was added to the HMX-PDA solution, and the reaction mixture was then stirred at room temperature for 16 h. The solution was then centrifuged and thoroughly washed with a PBS solution (10 mM, pH 7.0) at 5000 rpm once, followed by ultrasonic dispersion in 20 mL of PBS solution (10 mM, pH 7.0) for further use.

The preparation of the HMX@Al@CuO nanoenergetic composite was as follows. The PDA-peptide-formed HMX@Al@CuO nanoenergetic composite was synthesized in the following manner. The Al and CuO NPs were assembled on the HMX-PDA-SH-25-NH_2_ via the close interaction of the SH-25-NH_2_ coated on the HMX. The Al (45 mg) and CuO (45 mg) were separately dispersed in 15 mL of PBS solution (3 mg/mL, 10 mM, pH 7.0) and sonicated at 100 W for 5 min (with 2 s pulses separated by 1 s) to achieve a homogenous dispersion. The Al, CuO, and HMX-PDA-SH-25-NH_2_ dispersions were then mixed at a volume ratio of 33:92:250, followed by stirring at ambient temperature for 1 h. The product was centrifuged and then washed with DI water. The HMX@Al@CuO nanoenergetic composite was formed after two days of drying at 30 °C. The equivalence ratio (Φ) of the HMX@Al@CuO composite was fixed at 1.6 with the existence of the alumina shell. HMX was introduced into the HMX@Al@CuO composite at the ratio of 50 wt%. The mass contents of the PDA and SH-25-NH_2_ were negligible.

The physically mixed sample (HMX-Al-CuO) was synthesized using the previously described procedure without the addition of DA-HCl, and the SH-25-NH_2_ peptide aqueous solution was substituted with a Tris-HCl solution (10 mM, pH 8.5).

The PDA mixed sample (HMX-PDA-Al-CuO) was produced using the aforementioned procedure except that the SH-25-NH_2_-peptide aqueous solution was replaced with a Tris-HCl solution (10 mM, pH 8.5).

### 2.3. Characterization

A thermogravimetric analysis and differential scanning calorimetry (TGA/DSC 3+, Mettler Toledo, Greifensee, Switzerland) were utilized to examine the thermal properties of the samples from room temperature to 900 °C at heating rates of 5, 10, 15, and 20 °C/min in an argon (50 mL/min). For a typical measurement, around 2–4 mg of sample was placed into a 70 μL platinum crucible. Transmission electron microscopy (TEM, JEM-F200, JEOL, Tokyo, Japan) was employed to examine the morphological characteristics and elemental compositions of the samples. XPS (AXIS Ultra, Kratos Analytical Ltd., Manchester, UK) was utilized to identify the elemental state and chemical constituent of the synthesized sample. Fluorescence investigations were performed utilizing a fluorescence microscope (BX51, Olympus, Tokyo, Japan). To confirm the peptide conjugation [31], the SH-25-NH_2_ peptide was replaced by fluorescein isothiocyanate (FITC)-labeled SK-26.

## 3. Results

### 3.1. Morphological and Compositional Characterization

**Figure 1:**
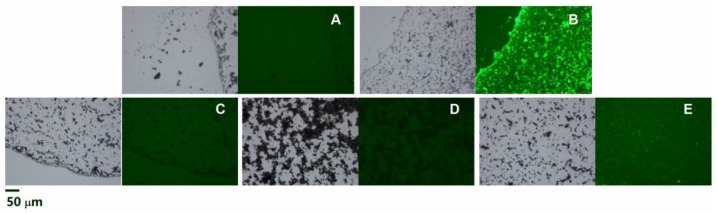
Fluorescence images of peptides incubated with (**A**) HMX-PDA, (**B**) HMX-PDA-SK-26, (**C**) HMX-Al-CuO, (**D**) HMX-PDA-Al-CuO, and (**E**) HMX-PDA-SK-26-Al-CuO. The white images were observed on a bright field and the blackish-green images were observed on a dark field.

The stable immobilization of the SH-25-NH_2_ peptide onto the HMX was crucial for the self-assembly of the HMX, Al, and CuO. Therefore, the SH-25-NH_2_ peptide should preferably be covalently linked to the HMX for stable linkage. The immobilization of the SH-25-NH_2_ peptide was explored using FITC-labeled SH-25-NH_2_ (SK-26). Figure 1 shows fluorescence images of the sample surfaces. Before the SK-26 coating, there was almost no fluorescent signal on the HMX-PDA surfaces. In contrast, after the modification with SK-26 on the HMX-PDA, a stronger fluorescence was observed on the HMX-PDA-SK-26 surfaces. HMX, CuO, and Al exhibited no fluorescence in the absence of an FITC peptide (Figure 1C,D). After the HMX-Al-CuO nanoenergetic composite was assembled via HMX-PDA-SK-26, the fluorescence was slightly reduced (a decrease in the content of HMX-PDA-SK-26 in HMX-PDA-SK-26-Al-CuO) but remained visible. All results support the idea that the peptide was covalently linked to the PDA coating.

**Figure 2:**
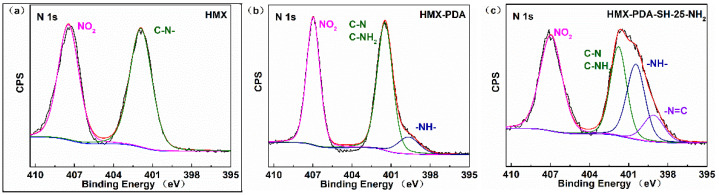
Typical high-resolution XPS N 1s spectra for (**a**) HMX, (**b**) HMX-PDA and (**c**) HMX-PDA-SH-25-NH_2_.

**Figure 3:**
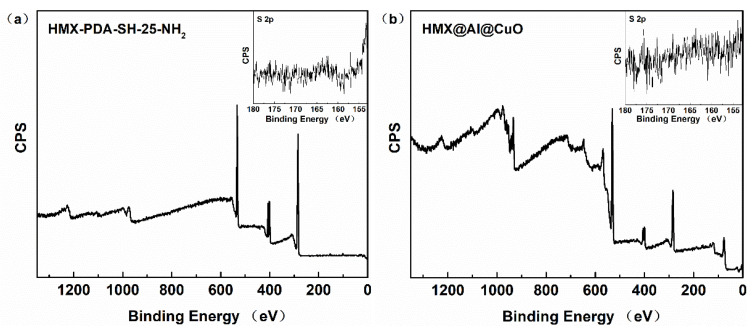
XPS characterization of (**a**) HMX-PDA-SH-25-NH_2_ and (**b**) HMX@Al@CuO.

Changes in the surface chemical compositions of the HMX, HMX-PDA, and HMX-PDA-SH-25-NH_2_ were monitored using XPS. The N 1s high-resolution XPS spectra are shown in Figure 2. The high-resolution N 1s spectrum of the HMX was deconvoluted into two curves with binding energies at 401.9 eV (C-N-) and 407.5 eV (-NO_2_) [22]. The presence of a PDA layer on the surface of the HMX-PDA after the PDA coating was further demonstrated by the formation of new N 1s peaks, which were attributed to -NH- at around 399.7 eV in the N 1s spectrum [17]. Compared with that of HMX-PDA, the new peak assigned to -N=C was observed after the grafting of the SH-25-NH_2_ peptide due to Schiff’s base or Michael addition reactions. Furthermore, the S 2p signal was discovered in the XPS scans of HMX-PDA-SH-25-NH_2_ and HMX@Al@CuO, which originated from the SH-25-NH_2_ (Figure 3). The surface characterizations demonstrate that the SH-25-NH_2_ peptide was successfully immobilized onto the HMX substrates.

**Figure 4:**
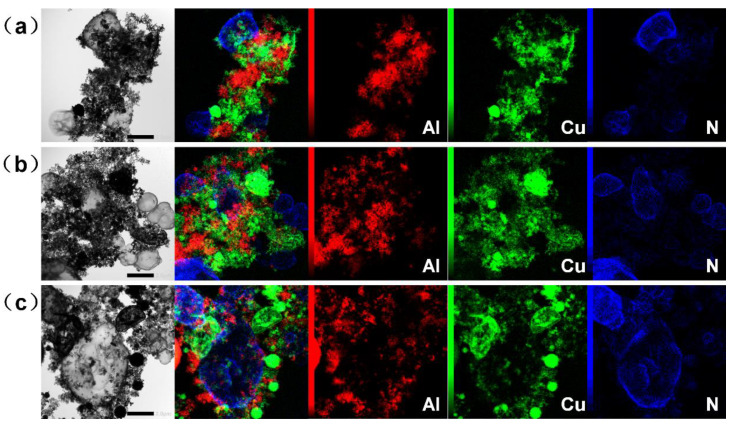
TEM and EDS mapping images of (**a**) HMX-Al-CuO, (**b**) HMX-PDA-Al-CuO, and (**c**) HMX@Al@CuO. Scale bars are 2 µm.

The arrangement of the HMX, Al, and CuO NPs, as well as their proximity to one another, played a significant influence on the energy release efficiency. As illustrated in Figure 4, the HMX-Al-CuO, HMX-PDA-Al-CuO, and HMX@Al@CuO micromorphologies were studied via TEM in combination with EDX. The three samples exhibited distinct dispersion and mixing characteristics. It was widely known that nitramine explosives are extremely sensitive to electron beams [3]. The HMX crystals appear to have small bubbles in Figure 4.

The TEM image of the HMX-Al-CuO and HMX-PDA-Al-CuO (Figure 4a,b) showed that both the Al and CuO were randomly distributed, and only small amounts of them were attached to the HMX surface. The respective EDS results from the Al and Cu elements indicate that their agglomerations were relatively severe. For HMX@Al@CuO (Figure 4c), the Al and CuO were self-assembled onto the HMX surface by the SH-25-NH_2_ peptide, which exhibited close contact with the HMX. In addition, these EDS mapping images demonstrate that HMX@Al@CuO demonstrates the best dispersion among the three types of samples examined in this investigation.

Based on these findings, it was determined that PDA-SH-25-NH_2_ played a crucial role in strengthening the interfacial contacts between HMX, Al, and CuO and improving their dispersion.

### 3.2. Thermal Analysis

**Figure 5:**
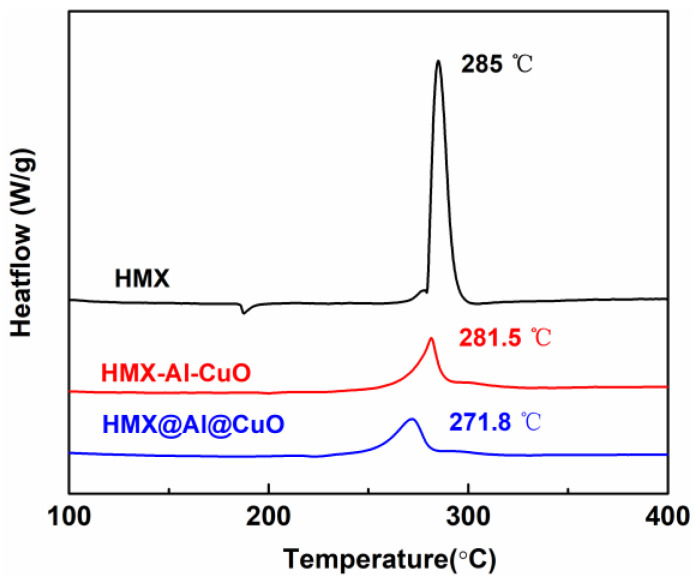
DSC curves at 10 °C /min for HMX, HMX-Al-CuO, and HMX@Al@CuO.

To determine the effects of Al and CuO on the performance of the HMX, DSC was conducted with the samples at the heating rate of 10 °C/min. From Figure 5, for HMX, the first endothermic peaks appearing at 188 °C could be attributed to the HMX (as the crystal changed from β type to δ type), while they disappeared for the HMX@Al@CuO. HMX was introduced into HMX@Al@CuO at a 50 wt% ratio, so the endo-peak of the phase transition of HMX could be too small to be attributed to a certain temperature.

**Figure 6:**
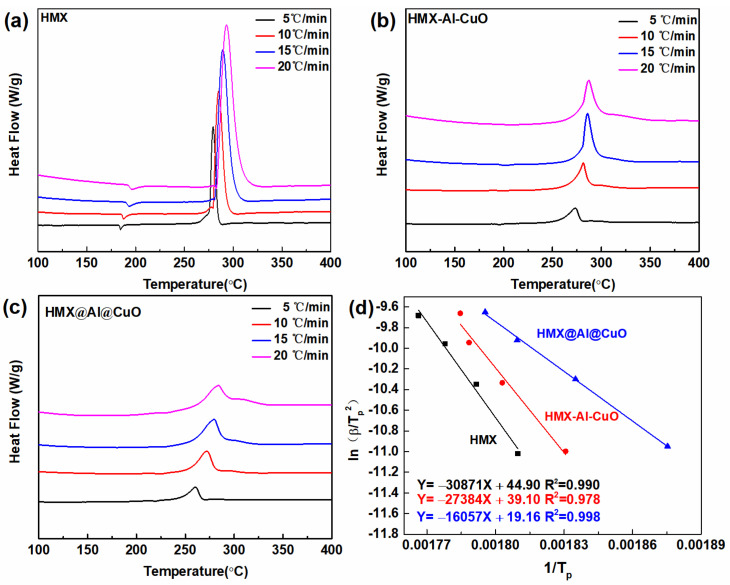
DSC curves at different heating rates for (**a**) HMX, (**b**) HMX-Al-CuO, and (**c**) HMX@Al@CuO; (**d**) linear fitting of ln (β/T_p_^2^) plotted against 1/T_p_.

Figure 6 and Table 1 displays the DSC findings for the HMX, HMX-Al-CuO, and HMX@Al@CuO at various heating rates (5.0, 10.0, 15.0, and 20.0 °C/min). As the heating rate increased, the HMX exothermic decomposition temperature peaks shifted to the right. The peak temperatures of the HMX@Al@CuO with different heating rates were 260.1 °C, 271.8 °C, 279.5 °C, and 283.8 °C, which were lower than those of raw HMX (279.4 °C, 285.0 °C, 289.3 °C, and 293.0 °C). To further illustrate the difference in the chemical reaction activity of the HMX in the HMX@Al@CuO, the E_a_ of the HMX was calculated using the Kissinger technique [32]:

(1) $$\ln\left(\frac{\beta }{T_{p}^{2}}\right)=-\frac{E_{a}}{RT_{p}}+\ln\left(\frac{AR}{E_{a}}\right)$$

where β represents the value of the linear heating rate (°C/min), R is the universal gas constant (8.314 J·mol^−1^·K^−1^), A denotes the pre-exponential factor (s^−1^), and T_p_ represents the exothermic peak temperature, K. The E_a_ values were determined to be 133.5 kJ/mol, 227.7 kJ/mol, and 256.7 kJ/mol for the HMX@Al@CuO, HMX-Al-CuO, and HMX, respectively. The decrease in the HMX’s activation energy after integrating with Al-CuO could be attributed to the catalyst effect of the Al-CuO nanothermite [5]. Research suggested that a nanosize metal size could provide the hotspots and physical adsorption sites to accelerate the decomposition of the HMX [33]. The determined E_a_ value for the HMX@Al@CuO was significantly lower than those for HMX-Al-CuO and HMX. The HMX@Al@CuO, Al, and CuO were self-assembled on the surface of the HMX by the SH-25-NH_2_ peptide, which demonstrated close contact with the HMX, resulting in a reduced E_a_ and therefore an enhanced reactivity.

## 4. Conclusions

In this study, an HMX@Al@CuO nanocomposite was synthesized for the first time using PDA-peptides. The integration of HMX with Al-CuO exploited the strong adhesion of PDA and the specific recognition of the SH-25-NH_2_ peptide to establish a close relationship between the explosive and the nanothermite. The E_a_ for the decomposition of the HMX@Al@CuO decreased by 48% when compared to the E_a_ for the decomposition of the HMX material. These results demonstrated that the use of PDA-peptides in the integration of explosives with nanothermites to form nanostructured energetic composites displays great potential for the environmentally friendly fabrication of nanostructured energetic composites with high levels of selectivity under mild reaction conditions. This study’s strategy can be applied to the fabrication of various nanostructured energetic composites with great performances.

## Figures and Tables

**Table 1:** Peak temperatures of different samples.

Samples	T_p_ (°C)			
	5 °C/min	10 °C/min	15 °C/min	20 °C/min
HMX	279.4	285.0	289.3	293.0
HMX-Al-CuO	273.1	281.5	286.0	287.2
HMX@Al@CuO	260.1	271.8	279.5	283.8

## Data Availability

The data presented in this study are available from the corresponding author upon reasonable request.
